# Evidence That Mast Cells Are Not Required for Healing of Splinted Cutaneous Excisional Wounds in Mice

**DOI:** 10.1371/journal.pone.0059167

**Published:** 2013-03-27

**Authors:** Allison C. Nauta, Monica Grova, Daniel T. Montoro, Andrew Zimmermann, Mindy Tsai, Geoffrey C. Gurtner, Stephen J. Galli, Michael T. Longaker

**Affiliations:** 1 Hagey Laboratory for Pediatric Regenerative Medicine, Division of Plastic and Reconstructive Surgery, Department of Surgery, Stanford University School of Medicine, Stanford, California, United States of America; 2 Oregon Health and Sciences University, Department of Surgery, Division of Plastic and Reconstructive Surgery, Portland, Oregon, United States of America; 3 University of California San Francisco School of Medicine, San Francisco, California, United States of America; 4 Department of Pathology, Stanford University School of Medicine, Stanford, California, United States of America; 5 Department of Microbiology and Immunology, Stanford University School of Medicine, Stanford, California, United States of America; Leiden University Medical Center, The Netherlands

## Abstract

Wound healing is a complex biological process involving the interaction of many cell types to replace lost or damaged tissue. Although the biology of wound healing has been extensively investigated, few studies have focused on the role of mast cells. In this study, we investigated the possible role of mast cells in wound healing by analyzing aspects of cutaneous excisional wound healing in three types of genetically mast cell-deficient mice. We found that C57BL/6-*Kit^W-sh/W-sh^*, WBB6F_1_-*Kit^W/W-v^*, and *Cpa3-Cre; Mcl-1^fl/fl^* mice re-epithelialized splinted excisional skin wounds at rates very similar to those in the corresponding wild type or control mice. Furthermore, at the time of closure, scars were similar in the genetically mast cell-deficient mice and the corresponding wild type or control mice in both quantity of collagen deposition and maturity of collagen fibers, as evaluated by Masson’s Trichrome and Picro-Sirius red staining. These data indicate that mast cells do not play a significant non-redundant role in these features of the healing of splinted full thickness excisional cutaneous wounds in mice.

## Introduction

Successful wound healing involves coordinated interactions between many different cell types. During the three overlapping phases of wound healing–inflammation, proliferation, and remodeling–cells are recruited to the wound bed to interact with resident cells in order to replace lost or damaged tissue and restore the skin’s integrity [1]. Although certain cell types have been extensively researched for their roles in tissue repair [2], [3], [4] the role of the mast cell has not been fully defined.

Mast cells contribute importantly to allergic and inflammatory responses, and have been implicated as key participants in the tissue remodeling associated with some of these responses [5]. Mast cells also have been proposed to have a role in several aspects of host responses to tumors [6], [7], [8]. In addition, there has been considerable speculation that mast cells can play important roles in wound healing, particularly in the early phases, when inflammation and angiogenesis allow clearance of debris and the delivery of nutrients to the wound bed [7], [9], [10], [11], [12], [13], [14], [15], [16], [17], [18], [19]. For example, it has been suggested that mast cell activation, resulting in degranulation and release of histamine and the synthesis and secretion of prostaglandin D_2_, can both promote repair [9], [15] and contribute to the formation of normal and pathological scarring [14], [19]. Indeed, mast cells can produce many growth factors with the potential to contribute to wound healing through effects on blood vessels [7], [20], [21], [22], as well as other products that can have complex effects on fibroblast proliferation and function [14], [17], [23], [24], [25].

A few studies have attempted to use mouse models to shed light on the role of mast cells in cutaneous wound repair [10], [11], [12], [19]. In two studies which analyzed the healing of cutaneous excisional wounds in adult mice, the rates of wound closure were not different in genetically mast cell-deficient WBB6F_1_-*Kit^W/W-v^* mice and the corresponding wild type mice [10], [11]. In another study, the closure of cutaneous excisional wounds was delayed in mast cell-deficient WBB6F_1_-*Kit^W/W-v^* mice vs. wild type mice during the first 6 days after wounding, but the wounds eventually closed at the same time as those in wild type mice [12]. Finally, Wulff *et al.* [19] reported that the scarring associated with full thickness cutaneous wounds examined 7 or 10 days after wounding on fetal day 18 was less in WBB6F_1_-*Kit^W/W-v^* mice than in wild type mice.

However, the results of such work need to be interpreted in light of evidence that there can be important differences in the features of wound healing among different animal species. For example, seventy to eighty percent of cutaneous excisional wound healing in mice occurs via skin contraction, whereas in humans the contribution of contraction is negligible [26]. We attempted to quantify the role of mast cells in features of cutaneous excisional wound closure by studying closure kinetics and scar formation in a mouse model that closely approximates physiological repair of cutaneous wounds in humans [26]. This model is based on the placement of a rubber washer that acts as a splint to minimize wound contraction in an animal, such as the mouse, that has loose skin and a skeletal muscle layer, the panniculus carnosus, in its subcutaneous tissue [26].

Using this model, we compared features of healing of such splinted cutaneous excisional wounds in three different types of mice that have profound genetically-determined reductions in the number of skin mast cells, and in the corresponding wild type or control mice. We analyzed the mast cell-deficient mice that have been used most often for studies of the roles of mast cells in biological responses, and in all of the prior studies of mouse cutaneous wound healing, namely (WB/ReJ-*Kit^W/+^* X C57BL/6J-*Kit^W-v/+^*)F_1_ (i.e., WBB6F_1_) WBB6F_1_-*Kit^W/W-v^* mice (18). WBB6F_1_-*Kit^W/W-v^* mice are markedly mast cell deficient in the skin and other anatomical sites due to loss of function mutations in the coding region of c-*kit* [27], which encodes KIT, the receptor for stem cell factor [28], [29]. By 6 to 8 weeks of age, WBB6F_1_-*Kit^W/W-v^* mice possess less than 1% the number of skin mast cells of their wild type counterparts, and have virtually no mast cells in many other organs [30]. Although this degree of mast cell deficiency is favorable for the study of mast cell function, the c*-kit* mutations of WBB6F_1_-*Kit^W/W-v^* mice result in multiple other potentially confounding phenotypic abnormalities that might influence analyses of wound healing, such as a macrocytic anemia [31], a reduction in levels of blood neutrophils [32], [33], [34], [35], [36] (a cell that also can contribute to wound healing [1]), and a predisposition of older WBB6F_1_-*Kit^W/W-v^* mice to spontaneous dermatitis [37].

We therefore also studied C57BL/6-*Kit^W-sh/W-sh^* mice, whose phenotype is altered by the effects of a large inversion mutation that affects the transcriptional regulatory elements upstream of the c*-kit* transcription start site [34]. *Kit^W-sh/W-sh^* mice exhibit striking mast cell depletion in most organ systems, including the skin [32], [33], [38]. However, unlike WBB6F_1_-*Kit^W/W-v^* mice, C57BL/6-*Kit^W-sh/W-sh^* mice are neither anemic [39] nor sterile [40]. Moreover, C57BL/6-*Kit^W-sh/W-sh^* mice have elevated levels of both neutrophils [33], [34], [35], [36] and platelets [33], [34], [35].

Toward the completion of our work in these two mast cell mutants, a third mast cell mutant mouse became available. We analyzed the features of excisional cutaneous wound healing in the newly described *Cpa3-Cre; Mcl-1^fl/fl^* mice, which do not have mutations affecting c-*kit* but instead are profoundly mast cell-deficient (and also have reduced numbers of basophils) because of the Cre-driven reduction in levels of the intra-cellular anti-apoptotic factor, Mcl-1, in two hematopoietic lineages that express carboxypeptidase 3 (CPA3), mast cells and basophils [41].

## Results

### Mast Cell-deficient Mice Close Excisional Wounds at Rates Similar to those of the Corresponding Control Mice

**Figure 1:**
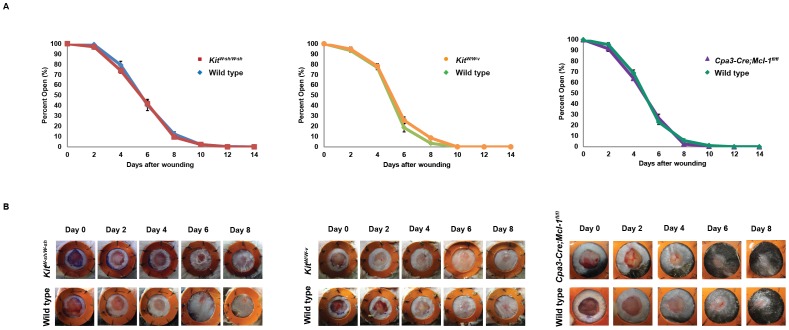
Kinetics of excisional wound healing in mast cell-deficient vs. corresponding control mice. (**A**) Splinted excisional wound healing in mast cell-deficient mutant vs. the corresponding control mice shown from left to right: C57BL/6-*Kit^W-sh/W-sh^* (*Kit^W-sh/W-sh^*) mice vs. C57BL/6-*Kit^+/+^* (wild type) mice, WBB6F_1_-*Kit^W/W-v^* (*Kit^W/W-v^*) mice vs. WBB6F_1_-*Kit^+/+^* (wild type) mice, and *Cpa3-Cre; Mcl-1^fl/fl^* vs. *Cpa3-Cre; Mcl-1^+/+^* (control) mice. (**B**) Representative images of wound healing from day 0 through day 8 in *Kit^W-sh/W-sh^* and corresponding wild type mice, in *Kit^W/W-v^* and corresponding wild type mice, and in *Cpa3-Cre; Mcl-1^fl/fl^* and corresponding control mice.

Eight *Kit^W-sh/W-sh^*, 10 *Kit^W/W-v^*, and 6 *Cpa3-Cre; Mcl-1^fl/fl^* mice and 8, 10 or 5 of the corresponding control mice were wounded with two 6 mm circular, splinted, full thickness excisional skin wounds per animal and followed for two weeks. Toluidine blue staining of skin tissue sections harvested at the time of wounding confirmed the markedly reduced numbers of dermal mast cells in the skin of the mast cell-deficient mice compared to those in the corresponding controls (**Fig. S1**). However, the three types of genetically mast cell-deficient mice re-epithelialized excisional wounds at rates that were indistinguishable from those of the corresponding control mice, with all mice healing excisional wounds by day 10 (**Fig. 1**). Wound healing is a highly evolved and efficient process in healthy animals; therefore, even small differences observed, if they are statistically significant, are considered meaningful. Our finding that the rates of wound healing, as assessed by re-epithelialization of the wound surface, were nearly identical in each of the three types of mast cell-deficient mice and the corresponding control mice suggests that the non-redundant contribution of mast cells to the rate of wound closure in this model is likely to be negligible.

### Mast Cell-deficient Mice Produce Scars Similar in Size and Collagen Content Compared to Control Mice

After establishing that there were no differences in the time to closure between excisional wounds created in mast cell-deficient mice and the corresponding control mice, we evaluated the quality of repair in terms of wound size, quantity of collagen, and collagen microarchitecture. Wounds were harvested at day 14 post-wounding, when all wounds exhibited full re-epithelialization. Wound tissue was harvested by excision of the scar and a rim of normal tissue. Skin histology showed similar scar sizes between the *Kit^W-sh/W-sh^*, *Kit^W/W-v^*, or *Cpa3-Cre; Mcl-1^fl/fl^* mice and the corresponding control mice. The differences we detected between the three types of mast cell-deficient mice and the corresponding control mice, whether in scar area (**Fig. S2A**), scar diameter (**Fig. S2B**), or dermal depth ratio (**Fig. S2C**), were not statistically significant. Taken together, these data suggest that, in healthy mice, scar size in this model is not markedly influenced by the presence or virtual absence of mast cells.

**Figure 2:**
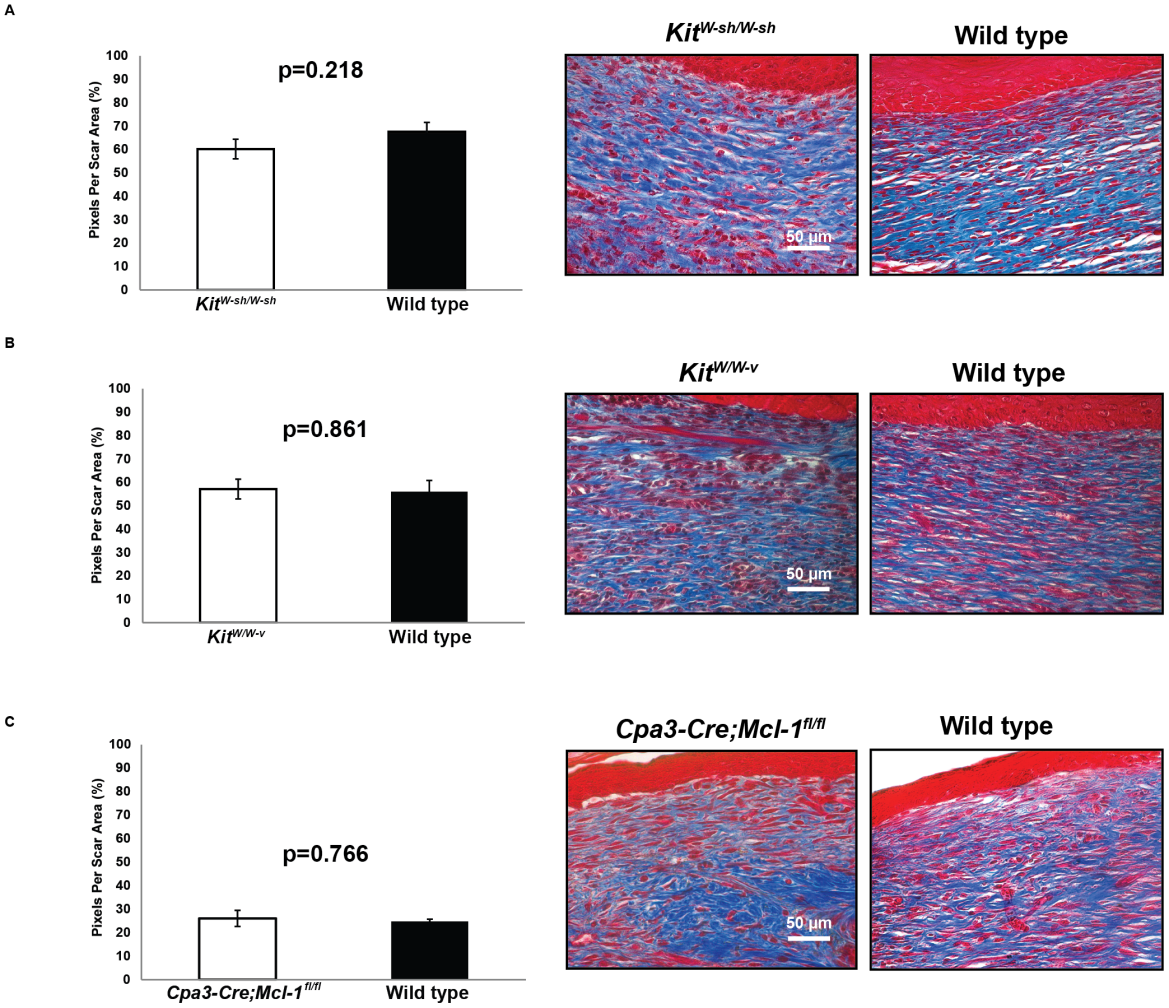
Measurements of collagen density in dermal scars in mast cell-deficient vs. corresponding control mice. Collagen density, quantified as average blue pixel density per scar area in skin harvested on day 14, in (**A**) C57BL/6-*Kit^W-sh/W-sh^* (*Kit^W-sh/W-sh^*) mice vs. wild type mice, (**B**) WBB6F_1_-*Kit^W/W-v^* (*Kit^W/W-v^*) mice vs. wild type mice, and (**C**) *Cpa3-Cre; Mcl-1^fl/fl^* vs. control mice. Representative images of Masson’s Trichrome staining used for the analyses are shown on right panel of **A–C**, with collagen staining blue. Scale bars: 50 µm.

Next, we evaluated scars for collagen density using Masson’s Trichrome staining. This stain identifies collagen present within the confines of the scar with a blue color, facilitating qualitative comparison of dermal collagen density between scars of equal size. No statistically significant differences in scar collagen density on day 14 after wounding were identified between excisional wounds created on any of the three types of mast cell-deficient mice and the respective control mice (**Fig. 2**). Finally, we assessed the microarchitecture of collagen fibers using Picro-Sirius red staining and polarization microscopy. This method demonstrated no significant mast cell-dependent differences in the quality of collagen in the wounds, in that the amounts of both mature (red-orange) and immature (green-yellow) collagen were similar between each type of mast cell-deficient mice and the respective wild type or control mice on day 14 post-wounding (**Fig. S3**).

## Discussion

Skin mast cells are derivatives of hematopoietic progenitors that mature in and then inhabit the dermis of uninjured skin, thereby taking residence at this important interface between the host and the environment. Accordingly, mast cells are often among the first responders to environmental stimuli experienced by the skin [5], [6], [16]. When tissue is injured, local mast cells can become activated, which in turn can trigger the cells to release a wide diversity of preformed and newly synthesized mediators that are thought not only to promote inflammation and vasodilation, but also to recruit other cells to the wound bed and to have effects on the phenotype and function of structural cells that reside in the skin [5], [13], [42].

Only a few studies have investigated cutaneous excisional wound healing in mast cell-deficient mice [10], [11], [12], [19]. All of these studies employed WBB6F_1_-*Kit^W/W-v^* mice and the corresponding wild type mice, and none of them investigated splinted wounds, such as we used in order to mimic more closely the healing of human excisional wounds. Thus, although some of the prior papers present data indicating that mast cells can contribute to certain features of skin wound healing, in none of these studies was it possible to assess wound re-epithelialization independently of wound contraction. In our splinted model of cutaneous excisional wound healing, we found that mast cells were neither essential nor detrimental to wound closure. Our findings thus are consistent with those of the wound healing studies performed by Egozi *et al.* [10] and Iba *et al.* [11], who reported similar wound healing kinetics in *Kit^W/W-v^* mice and the corresponding wild type mice. Although Weller *et al*. reported a significant impairment in wound healing kinetics in *Kit^W/W-v^* mutants compared to wild type mice between days 2 and 6, the overall kinetics of *Kit^W/W-v^* excisional cutaneous wound closure was similar to what we have reported, with both mutant and wild type mice virtually closing wounds by 10 days after wounding [12]. It is possible that differences in wound closure between *Kit^W/W-v^* and wild type mice noticed by Weller e*t al.* at early time points after wounding may have reflected direct or indirect effects of mast cells on wound contraction [43], a factor that does not come into play in our model because of the splinting of the wounds [26]. Our findings also suggest that the connection between mast cells and the promotion of fibrosis may not be as strong as previously proposed, as we found that scar size, scar collagen content, and collagen fiber structure in the three types of mutant mast cell-deficient mice we analyzed were similar to those in the corresponding control mice according to histological analysis.

Past reports are incongruent regarding any effects of mast cells on the quality of cutaneous wound repair. Iba *et al.* evaluated regional differences in collagen density in dermal scars at sites of excisional wounds in adult mice using Masson’s Trichrome staining and reported tighter and less interwoven collagen at the edges of day 15 wounds in *Kit^W/W-v^* versus wild type mice [11]. Wulff *et al.* reported that full thickness skin wounds produced significantly less scar tissue in *K^W/W-v^* E18 fetal mice than in wild type mice, as measured by scar width on Masson’s Trichrome-stained sections [19]. While each study supports a role for mast cells in the late stages of cutaneous healing in the models analyzed, we did not observe evidence for such a role for mast cells in our model. However, both the Iba *et al.* and Wulff *et al.* studies were performed in un-splinted models of wound healing [11], [19], and Wulff *et al*. exclusively studied fetal scarring [19]. These differences in the models used may have contributed to the differences between our data and theirs. Notably, our data are not completely incongruent with those of Wulff *et al.*, who reported no difference in the density of types I and III collagen, or the organization of collagen fibers using Picro-Sirius red stained E18 scar tissue [19].

Mast cell depletion in two of the mast cell-deficient mutants we studied resulted from mutations affecting c-Kit structure (in WBB6F_1_-*Kit^W/W-v^* mice) or expression (in C57BL/6-*Kit^W-sh/W-sh^* mice). Therefore, any differences noted in wound healing between these mice and the corresponding wild type mice might have reflected either the animals’ mast cell deficiency and/or other consequences of their mutations. Even though we did not detect statistically significant differences in our wound healing model between WBB6F_1_-*Kit^W/W-v^* or C57BL/6-*Kit^W-sh/W-sh^* mice and corresponding wild type mice, we examined the features of cutaneous excisional wound healing in *Cpa3-Cre; Mcl-1^fl/fl^* mice, whose deficiency in mast cells (and basophils) is due to the Cre-driven reduction in levels of the intra-cellular anti-apoptotic factor, Mcl-1, in two hematopoietic lineages that express carboxypeptidase 3 (CPA3): mast cells and basophils. Our findings in the *Cpa3-Cre; Mcl-1^fl/fl^* mice were similar to those in the *Kit^W/W-v^* and *Kit^W-sh/W-sh^* mutants, revealing no significant differences between mutant mice and corresponding control mice in the features of wound healing we quantified.

Although our study presents evidence that mast cells are not essential for cutaneous excisional wound closure, it is important to emphasize that wound repair is a complex process. We cannot rule out the possibility that mast cells importantly influence aspects of cutaneous wound healing that were not investigated in our study, or that mast cells can contribute to wound closure by directly or indirectly promoting the contraction of the wound [43], which was prevented in our model. Even in the aspects of wound healing we did measure, it is possible that mast cells provide some important functions that are also provided redundantly by other effector mechanisms, and therefore would not be detectable unless the other effector mechanisms also were impaired. Finally, we wish to emphasize that our work investigated a single, albeit informative, model of cutaneous excisional wound healing. There is evidence indicating that mast cells and certain mast cell-associated proteases can significantly influence host responses in other models of tissue injury, such as that associated with burns [14], [44], [45]. It is possible that mast cells also can contribute to other examples of pathological scar formation. Keloids and hypertrophic scars are characterized by raised, often painful and itchy scar tissue [14], and it is possible that these signs and symptoms reflect, at least in part, mast cell degranulation and histamine release in the wound bed [14], [19], [46], [47], [48].

However, our data, from experiments employing three different types of mice with severe, genetically-determined reductions in mast cell numbers, provide strong evidence that mast cells are not essential for providing non-redundant functions that importantly control the rate of re-epithelialization of cutaneous excisional wounds or the ultimate collagen deposition or maturation in the resulting scars. Further studies utilizing other models of tissue injury and healing may shed additional light on whether, to what extent, and in which settings mast cells can have non-redundant roles in the complex set of biological responses encompassed in the general term, wound healing.

## Materials and Methods

### Mouse Excisional Wound Healing Model

All mice were housed and provided food and water *ad libitum* in accordance with Stanford University animal care and use committee approved protocols. The Institutional Animal Care and Use Committee at Stanford University approved this study (IACUC protocol number 8638). Three types of genetically mast cell-deficient mutant mice were used for wounding experiments. *Kit^W-sh/W-sh^* mice on the C57BL/6J background and their wild type C57BL/6J-*Kit^+/+^* littermates [35] and *Cpa3-Cre; Mcl-1^fl/fl^* and the corresponding control littermates (*Cpa3-Cre+, Mcl-1^+/+^*) [41] were bred and maintained at the Stanford University Research Animal Facility. (WB/ReJ-*Kit^W/+^* X C57BL/6J-*Kit^W-v/+^*)F_1_ (hereafter: WBB6F_1_) WBB6F_1_-*Kit^W/W-v^* mice and the littermate wild type WBB6F_1_-*Kit^+/+^* mice were purchased from the Jackson Laboratory, Bar Harbor, ME (WBB6F_1_/J-*Kit^W/W-v^* compound heterozygote, order number 100410). The WBB6F_1_ mice were permitted to acclimate for at least one week in the vivarium before procedures were performed.

Eight female *Kit^W-sh/W-sh^* mice and 8 of the wild type controls, 10 female *Kit^W/W-v^* mice and 10 of the wild type controls, and 6 female *Cpa3-Cre; Mcl-1^fl/fl^* mutants and 5 of the corresponding female controls (*Cpa3-Cre+, Mcl-1^+/+^*) were used for *in vivo* wound healing experiments. All mice were 13 weeks old at the time of wounding, and a well-established, reproducible, splinted mouse excisional wound healing model was used as previously described [26]. In brief, induction of anesthesia was performed under isofluorane gas/oxygen mixture (2.5% isoflurane at 2 L per minute), followed by maintenance anesthesia at 1 L per minute. Dorsal fur was clipped, and, after depilation, the skin was prepped with povidone-iodine and alcohol. Two 6 mm full thickness circular wounds were placed through the panniculus carnosus on the dorsum of each animal at the same level, approximately 6 mm below the ears and 4 mm lateral to the midline. Two circular silicone 12 mm diameter stents (Invitrogen, Carlsbad, CA) were placed around the perimeter of the wound and secured in place first with glue, followed by 8 single interrupted Ethilon 6–0 sutures per stent (eSutures.com, Mokena, Illinois). Wounds were dressed with sterile Tegaderm™ dressing (3 M Healthcare, St. Paul, MN). The dressing was changed every other day under anesthesia until full wound closure. Digital photographs were taken at the time of surgery and every other day postoperatively until wounds were completely re-epithelialized and filled with new tissue. Wound area was quantified using ImageJ software (NIH, Bethesda, MD) and expressed as a ratio of wound circumference to silicone stent circumference. Percent closure was expressed as a ratio of the ratio at a given time point divided by the ratio on day 0×100%.

### Staining and Quantification of Mast Cells

At the time of wounding, the excised tissue removed from the skin to create the wound was fixed in 4% paraformaldehyde, processed routinely, and embedded in paraffin. Eight µm sections were cut, and mast cells were stained metachromatically with 0.1% toluidine blue, pH 1 and counted on an Olympus BX60 microscope equipped with an indexed grid at 20× magnification, by an observer not aware of the identity of individual sections. Numbers of mast cells were recorded according to area (mm^2^) in 4 to 6 consecutive fields along the length of tissue section, and 4 tissue sections were evaluated per sample. For each sample, mast cell numbers per mm^2^ were averaged, and the mean of all samples within a group (n = two tissue samples per mouse) was calculated to determine average number of mast cells per mm^2^.

### Staining and Quantification of Scar Size

At day 14 postoperatively, mice were sacrificed by inhalation of CO_2_, and the wounds were excised *en bloc* with a rim of surrounding tissue and bisected. Wound halves were fixed in 4% paraformaldehyde, processed routinely, and embedded in paraffin. Eight µm sections were cut perpendicular to the epithelial surface through the entire scar. Every tenth section throughout the wound was stained using Hematoxylin and Eosin. Each section was visualized under light microscopy at 10× (Leica microscope, Leica DM 4000B) and photographed using the Leica DFC 500 camera (Leica, Allendale, NJ). Photographs were evaluated for dermal scar area (**Fig. S2A**, representative image), scar length at the epidermal surface (**Fig. S2B**, representative image), and dermal depth ratio (**Fig. S2C**, representative image); all values were quantified in pixels using Adobe Photoshop software (Adobe, San Jose, CA). Depth ratios (expressed herein as pixel ratios) were determined by taking the average of three measurements of scar depth (quantified in pixels) divided by an average of three measurements of unwounded dermal depth (quantified in pixels) (**Fig. S2C**, representative image). All sections (approximately 8 per tissue sample) were averaged to determine an index value for each category per tissue sample, and a mean was calculated for all samples within each mouse group (n = 16 wounds each for *Kit^W-sh/W-sh^* and corresponding wild type mice; n = 20 each for *Kit^W/W-v^* and corresponding wild type mice; n = 12 for *Cpa3-Cre; Mcl-1^fl/fl^* mutants and n = 10 for corresponding control mice). Quantification of the features of the sections was performed by an observer not aware of the identity of individual sections.

### Staining and Quantification of Scar Collagen Content

Masson’s Trichrome staining of formalin-fixed, paraffin-embedded, 8 µm sections cut perpendicular to the epithelial surface through the entire scar was performed using standard protocols. Digital photographs under 20× magnification were acquired as previously described using light microscopy. The volume of collagen was then calculated by dividing the amount of blue staining per area of scar (expressed in pixels) using Adobe Photoshop software (Adobe, San Jose, CA). Picro-Sirius red staining was performed according to the manufacturer’s protocol (IHC World, Woodstock, MD). Polarization microscopy images were acquired at 20× using a light microscope (Leica 5000B) equipped with a polarization filter and camera (Leica DFC 500). Adobe Photoshop software was used to quantify immature (yellow/green) collagen fibers per scar area and mature (red/orange) collagen fibers per scar area (expressed as a ratio of pixel density). Quantification of the features of the sections was performed by an observer not aware of the identity of individual sections.

## Supporting Information

Figure S1 **Mast cell numbers in unwounded skin of mast cell-deficient vs. corresponding control mice. (A)** All three types of mast cell-deficient mice had markedly diminished numbers of mast cells in the skin vs. the corresponding wild type or control mice, as demonstrated by toluidine blue staining (**p<0.01). **(B)** Representative images of toluidine blue-stained, formalin-fixed, paraffin-embedded skin show abundant dermal mast cells in the specimens from wild type or control mice (red arrows) and virtually no mast cells detectable in the dermis of the corresponding mast cell-deficient mutant mice. Scale bars: 200 µm (and, in insets, 50 µm).
(TIF)

Figure S2 **Skin scar measurements in mast cell-deficient vs. corresponding control mice.** **(A–C)** Average scar areas (**A**, top panel), average scar diameters (**B**, top panel), and average dermal scar depth (**C**, top panel) in C57BL/6-*Kit^W-sh/W-sh^* (*Kit^W-sh/W-sh^*) mice vs. C57BL/6-*Kit^+/+^* (wild type) mice (left), WBB6F_1_-*Kit^W/W-v^* (*Kit^W/W-v^*) mice vs. WBB6F_1_-*Kit^+/+^* (wild type) (middle), and *Cpa3-Cre; Mcl-1^fl/fl^* vs. their control (*Cpa3-Cre; Mcl-1^+/+^*) mice (right). The lower panel in **A** shows a representative image of an H&E-stained section with the scar area delineated (area within dashed lines). The lower panel in **B** is a representative image showing how the surface diameter was measured. The lower panel in **C** is a representative image showing three sample scar depths (black dashed double arrows) and three unwounded dermal depths (red double arrows), which were used to calculate the pixel ratio shown (i.e., pixels of scar/pixels of unwounded dermis). Scale bars: 200 µm. All scars were measured using H&E-stained, formalin-fixed, paraffin-embedded tissues.
(TIF)

Figure S3 **Measurements of collagen maturity in dermal scars in mast cell-deficient vs. corresponding control mice.** **(A)** Red-orange (mature; red bars) and green-yellow (immature; green bars) collagen fibers were quantified using Picro-Sirius red staining for collagen in scar tissue harvested from C57BL/6-*Kit^W-sh/W-sh^* (*Kit^W-sh/W-sh^*) vs. wild type (left) mice, WBB6F_1_-*Kit^W/W-v^* (*Kit^W/W-v^*) vs. wild type mice (middle), and *Cpa3-Cre; Mcl-1^fl/fl^* vs. control *Cpa3-Cre; Mcl-1^+/+^* mice (right). **(B)** Representative polarized filter images of Picro-Sirius Red staining from left to right. Scale bars: 50 µm.
(TIF)
