# Supplementary figures and images for: Evidence That Mast Cells Are Not Required for Healing of Splinted Cutaneous Excisional Wounds in Mice
Source: PLoS One. 2013 Mar 27;8(3):e59167. doi: 10.1371/journal.pone.0059167 (PMC3609818; doi:10.1371/journal.pone.0059167)

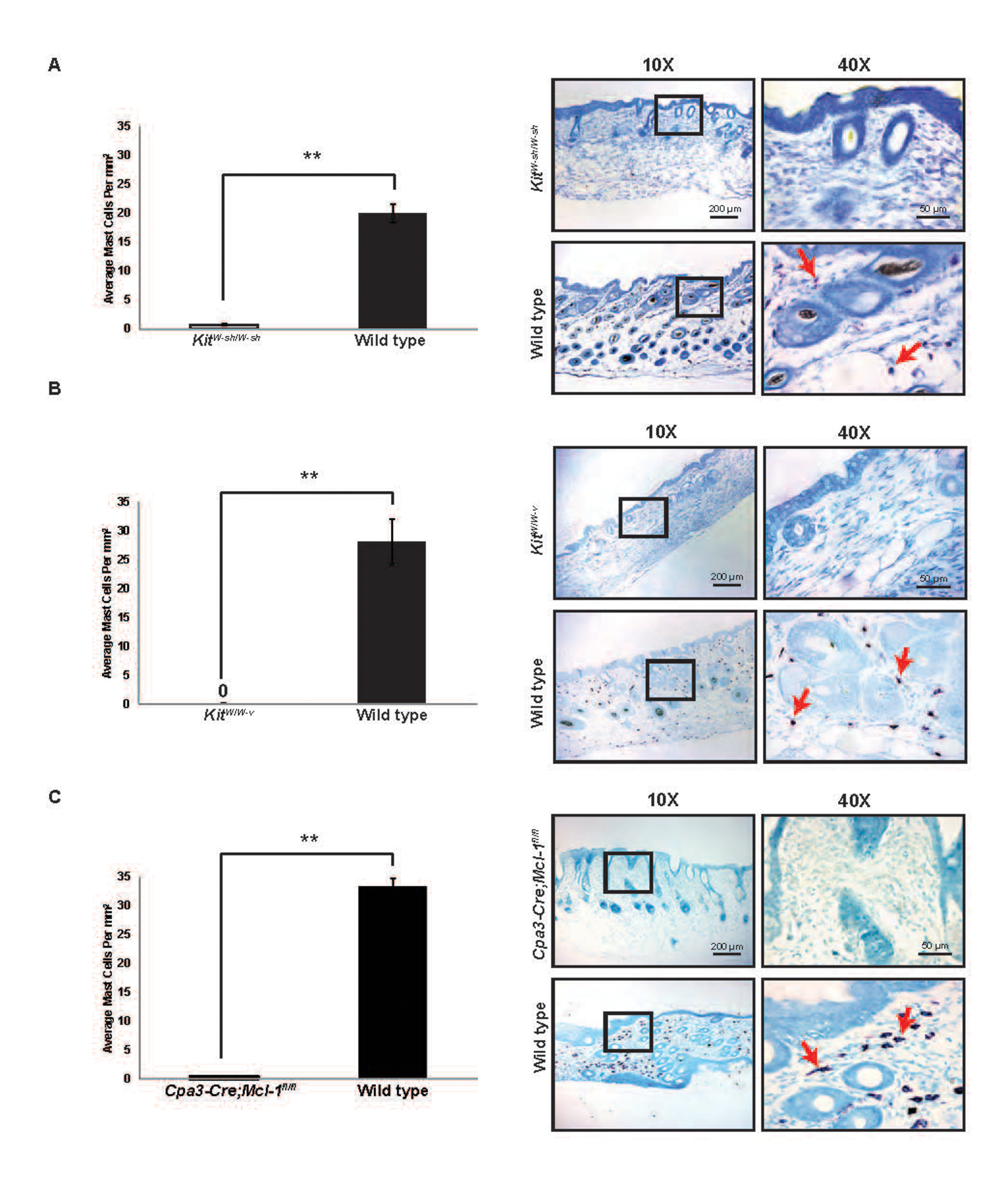

Supplement: Figure S1 — Mast cell numbers in unwounded skin of mast cell-deficient vs. corresponding control mice. (A) All three types of mast cell-deficient mice had markedly diminished numbers of mast cells in the skin vs. the corresponding wild type or control mice, as demonstrated by toluidine blue staining (**p<0.01). (B) Representative images of toluidine blue-stained, formalin-fixed, paraffin-embedded skin show abundant dermal mast cells in the specimens from wild type or control mice (red arrows) and virtually no mast cells detectable in the dermis of the corresponding mast cell-deficient mutant mice. Scale bars: 200 µm (and, in insets, 50 µm). (TIF) [file pone.0059167.s001.tif]

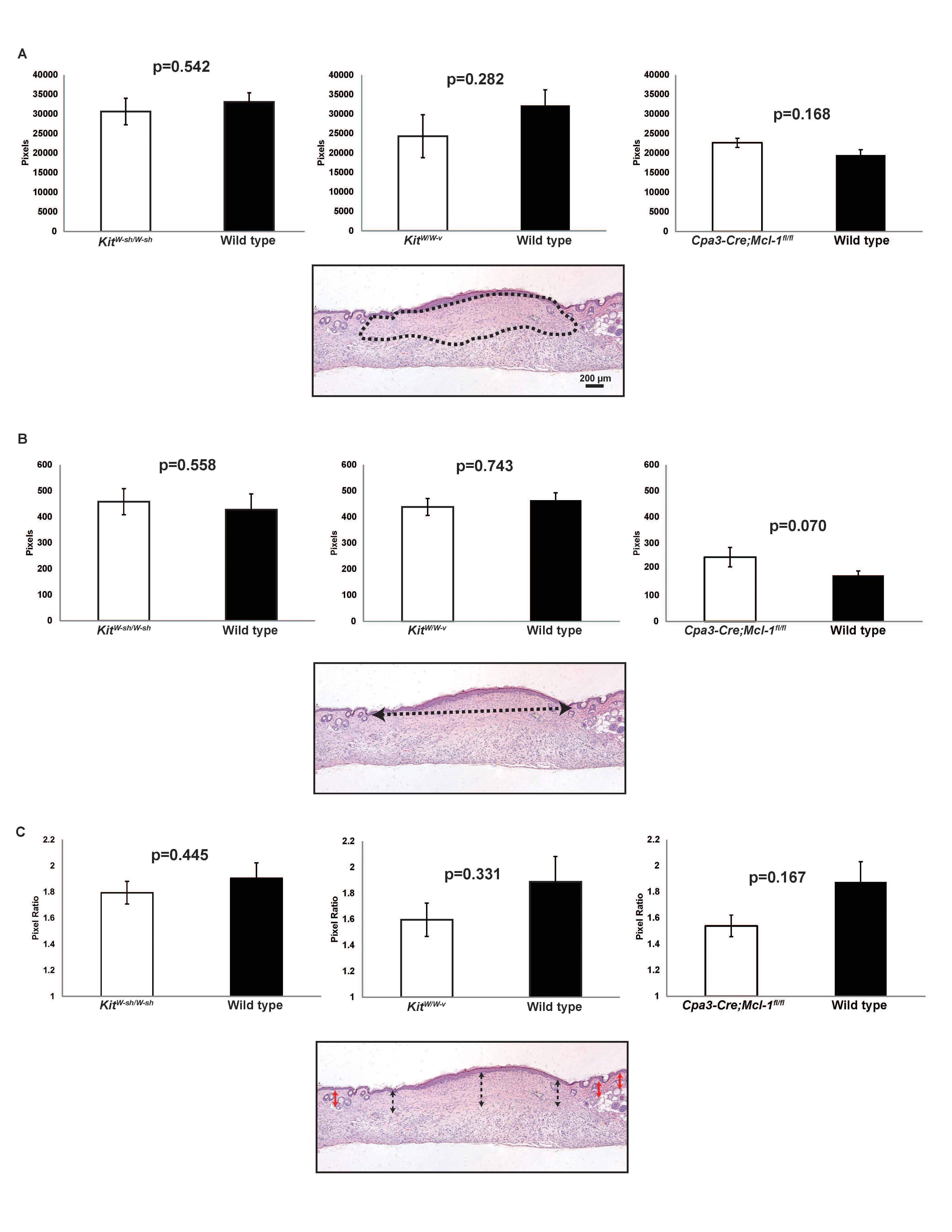

Supplement: Figure S2 — Skin scar measurements in mast cell-deficient vs. corresponding control mice. (A–C) Average scar areas (A, top panel), average scar diameters (B, top panel), and average dermal scar depth (C, top panel) in C57BL/6-KitW-sh/W-sh (KitW-sh/W-sh) mice vs. C57BL/6-Kit+/+ (wild type) mice (left), WBB6F1-KitW/W-v (KitW/W-v) mice vs. WBB6F1-Kit+/+ (wild type) (middle), and Cpa3-Cre; Mcl-1fl/fl vs. their control (Cpa3-Cre; Mcl-1+/+) mice (right). The lower panel in A shows a representative image of an H&E-stained section with the scar area delineated (area within dashed lines). The lower panel in B is a representative image showing how the surface diameter was measured. The lower panel in C is a representative image showing three sample scar depths (black dashed double arrows) and three unwounded dermal depths (red double arrows), which were used to calculate the pixel ratio shown (i.e., pixels of scar/pixels of unwounded dermis). Scale bars: 200 µm. All scars were measured using H&E-stained, formalin-fixed, paraffin-embedded tissues. (TIF) [file pone.0059167.s002.tif]

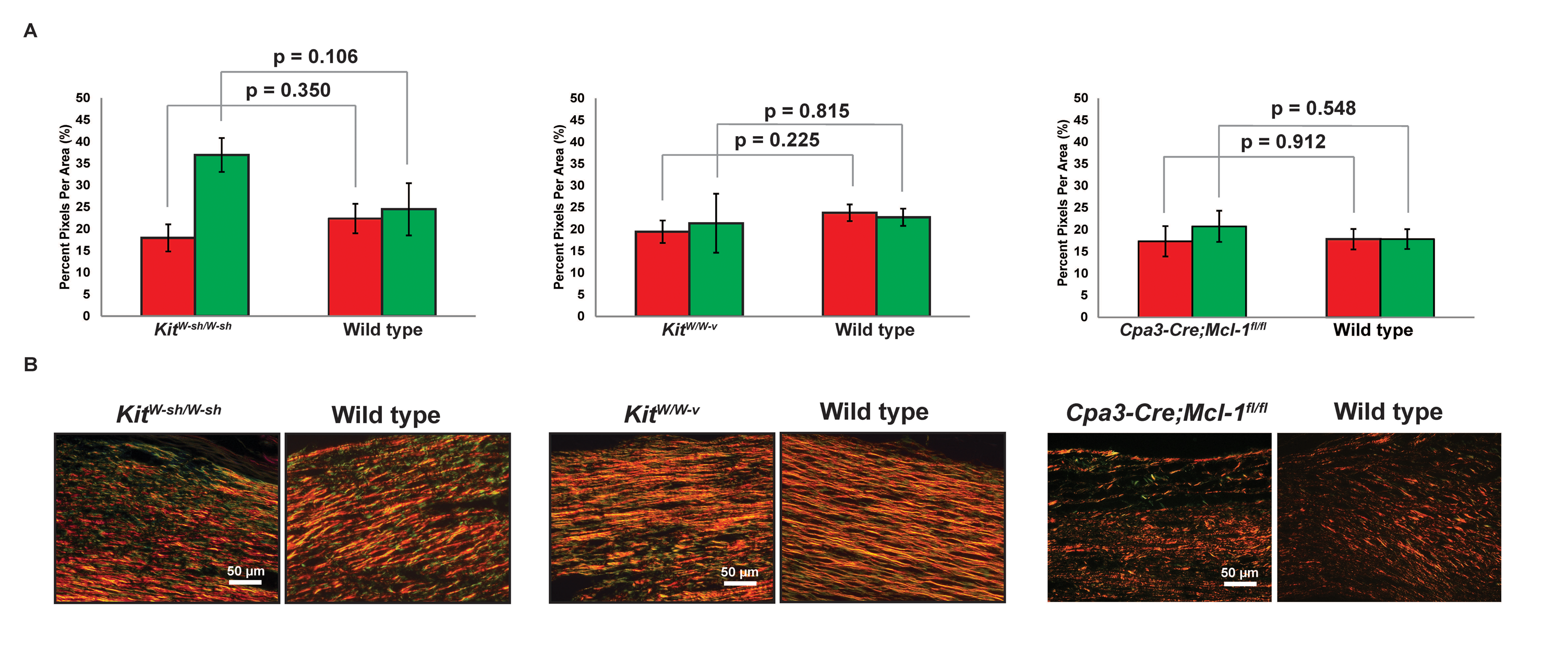

Supplement: Figure S3 — Measurements of collagen maturity in dermal scars in mast cell-deficient vs. corresponding control mice. (A) Red-orange (mature; red bars) and green-yellow (immature; green bars) collagen fibers were quantified using Picro-Sirius red staining for collagen in scar tissue harvested from C57BL/6-KitW-sh/W-sh (KitW-sh/W-sh) vs. wild type (left) mice, WBB6F1-KitW/W-v (KitW/W-v) vs. wild type mice (middle), and Cpa3-Cre; Mcl-1fl/fl vs. control Cpa3-Cre; Mcl-1+/+ mice (right). (B) Representative polarized filter images of Picro-Sirius Red staining from left to right. Scale bars: 50 µm. (TIF) [file pone.0059167.s003.tif]
